# Water Filters

**Published:** 1860-07

**Authors:** 


### WATER-FILTERS.

There is such a thing as going headforemost, of progressing backwards, and the use of water-filters in many cases is one of these, especially in New-York. The waters of the Mississippi, from St. Louis downwards, are thick with mud, and yet those who live along its banks and use it habitually, do not complain of its unhealthiness. If water passes through a common filter, its impurities are left in that filter; the next water which passes through has to pass through the detained impurities of the previous portion, and thus it goes on from day to day, for a new filter every day has not been advocated by any one. But it is the nature of the substances which water ordinarily holds in solution, to remain unchanged as long as they are covered or held in a plentiful amount of cool water, but when the water has been withdrawn, and they remain merely damp, they do, in the ordinary heat of summer, begin to undergo a process of destructive decay, they rot, and the very gases which they disengage in that process are speedily destructive of health and life, being the miasms which originate the deadly fevers on water-courses; if the gases be thus deadly, it may well be supposed that the more solid portions are not without their ill effect when taken into the stomach with water which is known to pass into the circulation within a few moments after it has been swallowed. That filtered water becomes changed in its character, is known by the familiar "flat" taste which belongs to it. Under this view of the case, it would be well to discard filters altogether, and not for the sake of getting rid of a few microscopic bugs and monsters, to take into the system instead, the deadly miasm which is generated in the sediment of all river-water, as soon as the water is removed and a summer temperature is applied to it, for then there is destructive decay; but no such thing can take place in the human stomach, although the sediment is swallowed in much larger quantities. These remarks do not apply to substances which may be thrown into the water and precipitate its impurities to the bottom, from which the water is at once poured off and used and the sediment thrown away; nor does it apply to any filter which may be thoroughly cleansed after each single filtering process. All filtrations yet known do take away from the water that fresh, sparkling, and refreshing quality which belongs to the bubbling spring, or is taken from the brim of "the moss-covered bucket which hangs in the well."

The "Double Globe Filter," patented in January last, has been brought to our notice since the above was put in type. It costs three dollars, can filter a gallon of water in five minutes; it does it nearly as fast as it runs from a "Croton" faucet, to which it is attached permanently or removed in half a minute. It is metallic, and, with care, will last a lifetime. This filter is thoroughly washed and cleansed in an instant by the easy crook of the finger; and if this cleansing is neglected, the water will not run. It is capable of universal application, it delivers the water with all its freshness, and seems to perform its office as perfectly as is necessary for all drinking and cooking purposes.

